# (Sialyl)Lewis Antigen Expression on Glycosphingolipids, *N*-, and *O*-Glycans in Colorectal Cancer Cell Lines is Linked to a Colon-Like Differentiation Program

**DOI:** 10.1016/j.mcpro.2024.100776

**Published:** 2024-04-25

**Authors:** Di Wang, Katarina Madunić, Oleg A. Mayboroda, Guinevere S.M. Lageveen-Kammeijer, Manfred Wuhrer

**Affiliations:** 1 Center for Proteomics and Metabolomics, Leiden University Medical Center, Leiden, The Netherlands; 2 Department of Cellular and Molecular Medicine, Copenhagen Center for Glycomics, University of Copenhagen, Copenhagen, Denmark; 3 Division of Analytical Biochemistry, Groningen Research Institute of Pharmacy, University of Groningen, Groningen, The Netherlands

**Keywords:** *N-/O*-glycans, glycosphingolipids, glycosyltransferases, transcription factors, CRC cell line

## Abstract

Alterations in the glycomic profile are a hallmark of cancer, including colorectal cancer (CRC). While, the glycosylation of glycoproteins and glycolipids has been widely studied for CRC cell lines and tissues, a comprehensive overview of CRC glycomics is still lacking due to the usage of different samples and analytical methods. In this study, we compared glycosylation features of *N*-, *O*-glycans, and glycosphingolipid glycans for a set of 22 CRC cell lines, all measured by porous graphitized carbon nano-liquid chromatography-tandem mass spectrometry. An overall, high abundance of (sialyl)Lewis antigens for colon-like cell lines was found, while undifferentiated cell lines showed high expression of H blood group antigens and α2-3/6 sialylation. Moreover, significant associations of glycosylation features were found between the three classes of glycans, such as (sialyl)Lewis and H blood group antigens. Integration of the datasets with transcriptomics data revealed positive correlations between (sialyl)Lewis antigens, the corresponding glycosyltransferase FUT3 and transcription factors *CDX1, ETS, HNF1/4A, MECOM,* and *MYB.* This indicates a possible role of these transcription factors in the upregulation of (sialyl)Lewis antigens, particularly on glycosphingolipid glycans, *via* FUT3/4 expression in colon-like cell lines. In conclusion, our study provides insights into the possible regulation of glycans in CRC and can serve as a guide for the development of diagnostic and therapeutic biomarkers.

## Highlights

• Glycosylation motifs are compared for a set of 22 colorectal cancer cell lines.
• For (sialyl)Lewis and H blood group antigens significant associations are found across the three glycan classes.
• Colon-like cell lines present high abundance of (sialyl)Lewis antigens.
• High expression of (sialyl)Lewis antigens might result from the regulation of FUT3.

## Graphical Abstract

**Figure:**
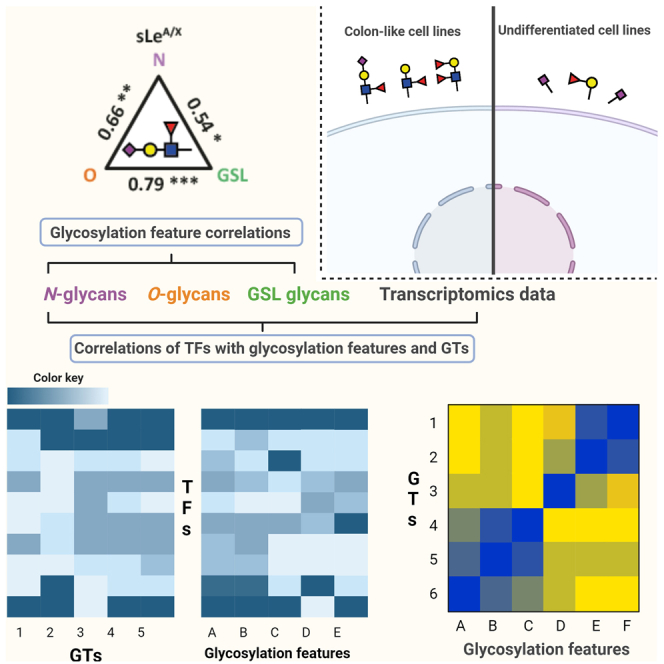

## In Brief

An integrated analysis of glycosylation features including (sialyl)Lewis structure and blood group antigens was performed across *N*-, *O*-glycans, and glycosphingolipid glycans. Colon-like lines exhibited abundant (sialyl)Lewis antigens, while undifferentiated lines expressed H blood group antigens and α2-3/6 sialylation. The observed associations across glycan classes along with correlations with glycosyltransferases and transcription factors suggest that certain transcription factors like CDX1 contribute to the upregulation of (sialyl)Lewis antigens on all three glycan classes *via* regulation of glycosyltransferases FUT3/4.

Based on the data from Global Cancer Statistics 2020, colorectal cancer (CRC) has become the third most commonly diagnosed cancer (10.0%) and the second leading cause of cancer death (9.4%) worldwide (1). Traditional treatments for cancer include chemotherapy, radiation, and surgery. Specific molecular targeting methods are increasingly implemented in clinical practice, including specific inhibitors (2) and monoclonal antibodies (3). Unfortunately, for a large part of the cases, these treatments turn out not to be effective due to tumor heterogeneity and detection at an advanced stage (4). Hence, new treatment strategies are urgently needed.

Glycosylation has shown to be a promising field for finding new biomarkers in diagnosis and specific targets for therapy, as an altered glycosylation profile has been related to the development and progression of cancer, such as tumor angiogenesis, invasion, and metastasis (5).Several factors contribute to the abnormal expression of glycosylation including (i) altered expression of glycosyltransferases (GTs) (6, 7, 8, 9), (ii) the changes in the activity of GTs (5), (iii) the mislocalization of GTs in the ER and Golgi apparatus (10, 11), and (iv) the availability and abundance of sugar donors (12).

Common cancer-associated glycosylation features include alterations in the level of fucosylation and sialylation (5, 13). Of which, the latter is involved in cell recognition, adhesion, and signaling (5). The upregulation of sialyl transferase ST6GAL1 results in overexpression of α2-6 sialylation and has been linked to changes in the adhesion of cancer cells to the extracellular matrix proteins like collagen, fibronectin, and laminin in colon cancer which contribute to metastasis and poor survival of CRC patients (14). A well-known sialylation feature is the Sialyl-Lewis X (sLe^X^) antigen [tetrasaccharide composed of a sialic acid (α2-3 linked), galactose (β1-4 linked), fucose (α1-3 linked), and an *N*-acetyllactosamine] which is a ligand for selectins which are vascular cell adhesion molecules involved in extravasation of cancer cells leading to formation of metastasis in secondary sites. Elevated expression of sLe^X^ was found to be associated with poor survival of CRC patients (15). Some well-established serological biomarkers for cancer detection, monitoring, and prognosis are carbohydrate antigens (CA) or glycoproteins such as sLe^A^ (also known as CA 19–9 and differs from sLe^X^ as the galactose is β1-3 linked and the fucose α1-4 linked) (16) and carcinoembryonic antigen (17, 18). However, the specificity of these biomarkers is limited as they are not only expressed in cancer cells but also in nonneoplastic and inflammatory diseases (19).

Many glycosylation features are shared between glycoproteins and glycolipids, and to gain better insights into the common features and GT isoenzyme specificities, a deeper exploration should be performed to investigate which of these shared features are correlated to CRC. Eventually this knowledge will aid to the discovery of specific tumor-associated glycans for diagnosis and targeted treatment of CRC.

On the basis of analysis of mutations and RNA and protein expression, CRC cell lines have been classified into two groups, which are colon-like cell lines with expression of gastrointestinal differentiation markers and undifferentiated cell lines characterized by upregulation of genes linked to epithelial–mesenchymal transition (EMT) (20). Mass spectrometry (MS) is a powerful tool to perform in-depth characterization of glycomic profiles and has been widely used to study the role of glycosylation in cancer, including CRC (21, 22, 23). Just recently, we examined the protein *N*- and *O*-glycosylation (22, 23) as well as glycosphingolipids (GSLs) glycosylation profiles (21) of CRC cell lines, using porous graphitized carbon nanoliquid chromatography-MS/MS. Striking differences were found between colon-like and undifferentiated cell lines for all three glycan classes. With regard to *O*-glycosylation, colon-like cell lines showed high expression of I-branched and sLe^A/X^ epitope-carrying glycans, while undifferentiated cell lines were characterized by high prevalence of truncated α2-6 core sialylated glycans, and some undifferentiated cell lines expressed high abundances of glycans with blood group antigens (A, B, and H) (23). As for *N*-glycans, colon-like cell lines presented a high expression of sulfation, (s)Le^A/X^, Le^B/Y^, antenna fucosylation, oligomannosidic, and hybrid-type *N-*glycans, while undifferentiated cell lines highly expressed phosphorylation, bisection, and α2-3 sialylation, as well as paucimannosidic *N*-glycans and *N*-glycans carrying (fucosylated) LacdiNAc (GalNAcβ1-4GlcNAc) (22). When it comes to GSL glycans, high expression of (s)Le^A/X^ and Le^B/Y^ antigens was found for colon-like cell lines while undifferentiated cell lines showed higher abundances of glycans with all blood group antigens (A, B, and H) (21).

In regard to GTs, ST6GALNAC1-4 has been reported to add sialic acid to *N*-acetylgalactosamine (GalNAc) of *O*-glycans (24), while ST6GALNAC5/6 seems to be involved in the sialylation of GSL glycans (25, 26). Regarding fucosylated glycans synthesized by a range of fucosyltransferases (FUT1-FUT11), previous studies have found that the expression of sLe^X^ is mainly regulated by FUT6 in breast cancer, while FUT7 plays an essential role in the upregulation of sLe^X^ in adult T cell leukemia cells (27, 28). However, it remains unclear which fucosyltransferase(s) contribute to the expression of Le antigens in CRC and to which extent the biosynthetic programs are shared between the three glycan classes.

In the present study, we performed an integrated analysis of commonalities and discrepancies in the expression of differentiation- and cancer-associated glycosylation features of CRC cell lines. Subsequently, we explored the association of CRC cell line glycosylation features with relevant GTs and transcription factors (TFs). Overall, our study provides novel insights into the potential (dys-)regulation of glycosylation in CRC across glycan classes, revealing glycosylation markers with potential diagnostic and therapeutic potential.

## Materials and Methods

Our recent studies provided in-depth comprehensive glycomic profiling (*N*-, *O*-glycans, and GSL glycans) of CRC cell lines (21, 22, 23). Glycomics data for CRC cell lines were retrieved from GlycoPOST (29): GPST000239 (GSL glycans) (21), GPST000035 (*O*-glycan) (23), and GPST000302 (*N*-glycan) (22). An overview of the included CRC cell lines is provided in supplemental Table S1. Taking advantage of the available data, glycans were assigned to different glycosylation features as indicated in supplemental Tables S2–S5 for *N*-, *O*-glycans, and GSL glycans, respectively. Only the CRC cell lines that had glycomics data for all classes available (*N*-, *O*-glycosylation, and GSLs glycosylation) were taken along. Multi-omics (DNA, RNA, and protein) datasets of CRC cell lines have been investigated and applied for CRC cell lines classifications. The transcriptomics data of CRC cell lines were retrieved from the Gene Expression Omnibus GSE97023 (20). Colon-like cell lines were characterized by the expression of gastrointestinal differentiation markers, and undifferentiated cell lines exhibited an upregulation of EMT pathway and transforming growth factor β signaling (20). Data analysis and visualization were conducted in “R” (version 4.2.1) with packages “tidyverse”, “Rcpm”, “pcaMethods”, “stringi”, “readxl”, “ggplot2”, “ggrepel”, “reshape2”, “tidyHeatmap”, and “corrplot”.

### Experimental Design and Statistical Rationale

We combined the *N*-, *O*-glycomic, and GSL glycomic data which all have been measured by porous graphitized carbon nanoliquid chromatography-MS/MS for the same set of CRC cell lines (21, 22, 23). More information about glycomic data can be found in supplemental Tables S2–S5. For integration, glycosylation traits and motifs determined within *N*-, *O*-glycomic, and GSL glycomic datasets were summed, thereby generating composite, integrated glycosylation traits. The transcriptomics data of CRC cell lines were obtained from Gene Expression Omnibus GSE97023 (20). For principal component analysis, a minimum positive number (0.00001) was used in case of missing data. Spearman correlations were conducted between glycosylation features of the three glycan classes as well as between glycosylation features with corresponding GTs and selected TFs.

## Results

**Fig. 1:**
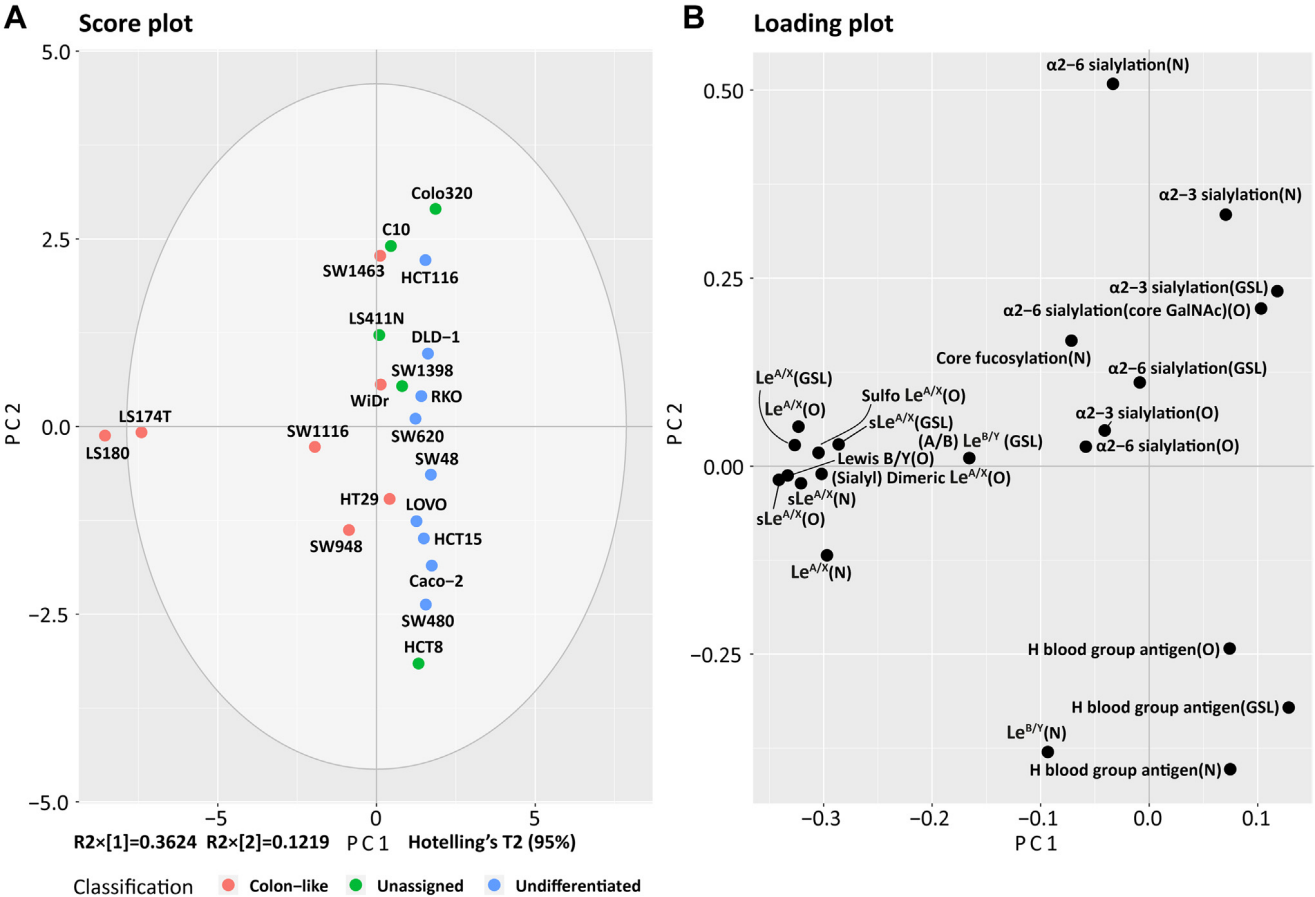
**Principle component analysis (PCA) of glycosylation features on *N*-, *O*-glycans, and GSL glycans in CRC cell lines.***A* and *B*, the score plot depicts the distribution of CRC cell lines colored by CRC cell line classifications (*A*), driven by the glycosylation features on *N*-, *O*-glycans, and GSL glycans displayed in the loading plot (*B*). Relative quantification of glycosylation features was used for PCA analysis. The top two principal components explain 48% of the variation within the data. Together with the third and fourth principal components, a variance of 69% was covered (supplemental Fig. S1). CRC, colorectal cancer; GSL, glycosphingolipid.

Integrated glycosylation features of CRC cell lines reflect the differentiation status, and CRC cell lines recapitulate the molecular alteration and pharmacogenomics of primary tumors and are therefore often used as preclinical models of CRC (20, 30, 31). To gain a comprehensive landscape of glycosylation of CRC cell lines and to explore the tumor-associated glycosylation features, the glycosylation features on *N*-, *O*-glycan, and GSL glycan were utilized and integrated based on relative quantification in each individual study (supplemental Table S6). Classifications of the CRC cell lines were demonstrated by principal component analysis (Fig. 1*A*) driven by the glycan class and specific glycosylation features (Fig. 1*B*).

Colon-like cell lines (LS180, LS174T, SW1116, WiDr, SW948, and HT29) clustered due to the expression of (s)Le^A/X^ and Le^B/Y^ (*N*-, *O*-glycan, and GSL glycans), sulfo Le^A/X^ (*O*-glycan), and (sialyl) dimeric Le^A/X^ (*O*-glycan) (Fig. 1). The highest abundance of Le^A/X^ on *N*-, *O*-glycan, and GSL glycans was found for cell line LS180 (6%, 4%, and 36%, respectively) and LS174T (5%, 5%, and 49%, respectively) (supplemental Fig. S2 and Table S6). Similar to Le^A/X^, the highest abundance of sLe^A/X^ structures was observed for LS180 and LS174T for all three glycan classes (supplemental Fig. S2 and Table S6). Notably, Le^B/Y^ epitopes on *O*-glycans were only expressed in cell lines LS180 and LS174T with relative quantification of 1.53% and 1.47%, while 0.38% and 0.22% of relative quantification of Le^B/Y^ epitopes on *N*-glycans in cell lines LS180 and LS174T was detected. The highest expression of Le^B/Y^ epitope on *N*-glycans was found in cell line HCT8 (unassigned). The highest abundance of (A/B) Le^B/Y^ (with the structure GalNAcα1-3(Fucα1-2) Galβ1-3/4(Fucα1-4/3) GlcNAc-R for A Le^B/Y^ and Galα1-3(Fucα1-2) Galβ1-3/4(Fucα1-4/3) GlcNAc-R for B Le^B/Y^) on GSL glycans was detected in the well-differentiated cell line SW1463 (supplemental Fig. S2 and Table S6).

In regard to sialylation, α2-3/6 sialylation was found on all three glycan classes and contributed to the grouping of undifferentiated cell lines HCT116, DLD-1, RKO, and SW620 as well as most unassigned cell lines Colo320, C10, LS411N, and SW1398 (Fig. 1). Colon-like cell line SW1463 revealed the highest α2-3 sialylation expression in the *N*-glycan class. HCT8 (unassigned regarding its differentiation status) clustered together with the undifferentiated cell lines SW48, LOVO, HCT15, Caco-2, and SW480 driven by the expression of blood group antigens on *N*-, *O*-glycan, and GSL glycans (Fig. 1). HCT8 was found to highly express blood group antigens on *N*-glycans with a relative abundance of 2%, and the highest expression of blood group antigens on *O*-glycans was found in the Caco-2 cell line (62%) (supplemental Fig. S2 and Table S6). In regard to the blood group antigens on GSL glycans, the highest abundance was detected in undifferentiated cell line LOVO (64%).

### Correlation of Glycosylation Features Between Glycan Classes

**Fig. 2:**
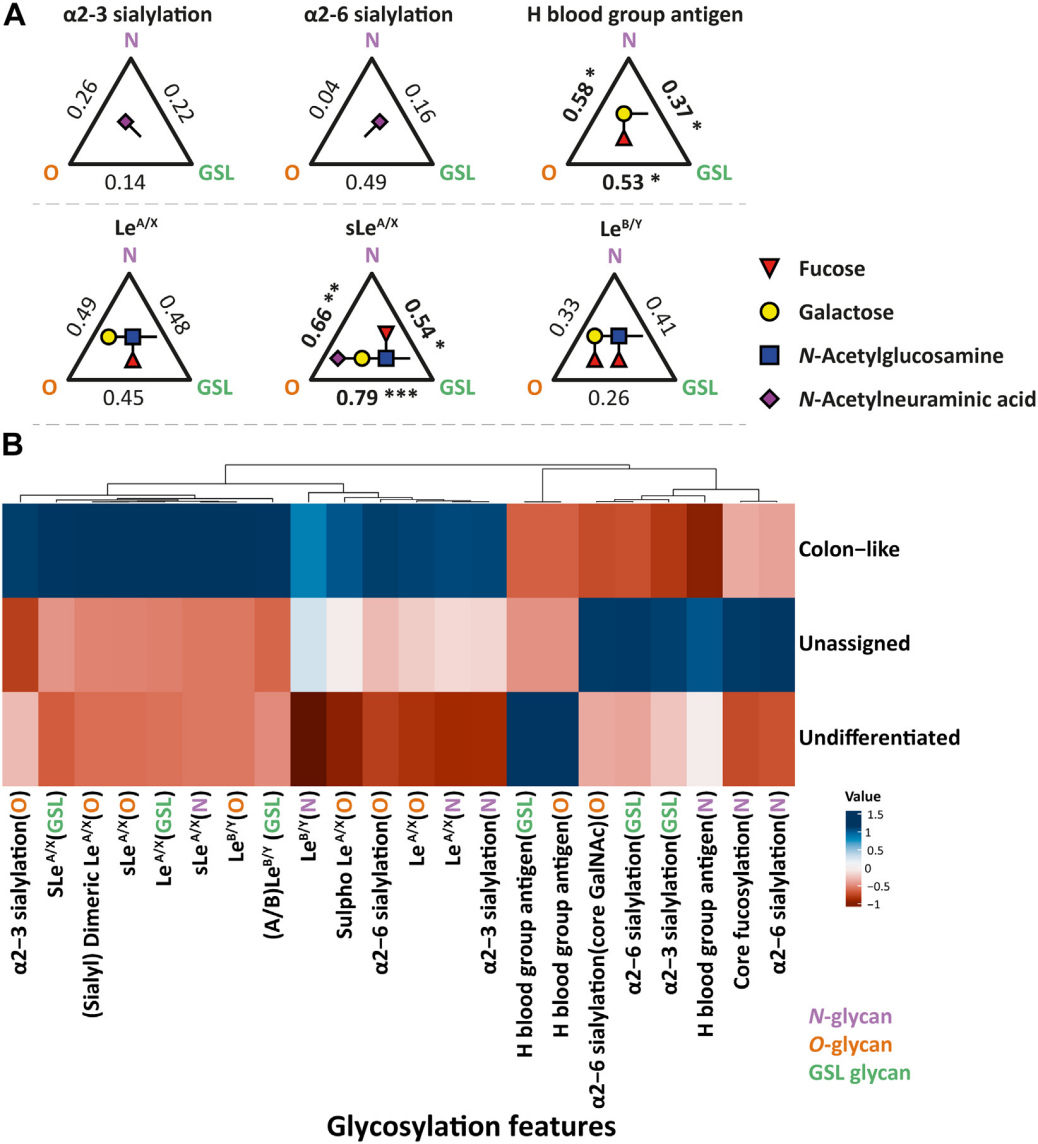
**Correlation of glycosylation features between three classes of glycans and distribution of glycosylation features on *N*-, *O*-glycans, and GSL glycans (bottom) in three CRC cell line classifications (right).***A*, Spearman correlations between glycosylation features of three glycan classes are visualized. Significant value is marked with ∗ (*p* ≤ 0.05), ∗∗ (*p* ≤ 0.01), and ∗∗∗ (*p* ≤ 0.001). *B*, glycosylation features of three classifications of CRC cell lines are compared. Relative abundances of each glycosylation feature on *N*-, *O*-glycans, and GSL glycans were used as input. For standardization, the mean was equaled to zero, and the standard deviation was equaled to 1. CRC, colorectal cancer; GSL, glycosphingolipid.

Next, the correlation of glycosylation features between the three glycan classes was explored. No significant correlations were observed for α2-3 and α2-6 sialylation among the three glycans classes (Fig. 2*A*). Significant correlations were revealed between all glycan classes for H blood group antigens as well as sLe^A/X^ antigens (Fig. 2*A*). Subsequently, we explored the glycosylation features for the different CRC cell line classifications (Fig. 2*B*). A rather high abundance of (s)Le^A/X^ and (A/B) Le^B/Y^ was observed for all three glycan classes in colon-like cell lines. Likewise, these cell lines showed high α2-3/6 sialylation on *O*-glycans and α2-3 sialylation on *N*-glycans. In contrast, blood group antigens on *O*-glycans and GSL glycans were highly expressed in undifferentiated cell lines (supplemental Table S7 and Fig. 2*B*).

### Correlation of Glycosylation Features and GTs in CRC Cell Lines

**Fig. 3:**
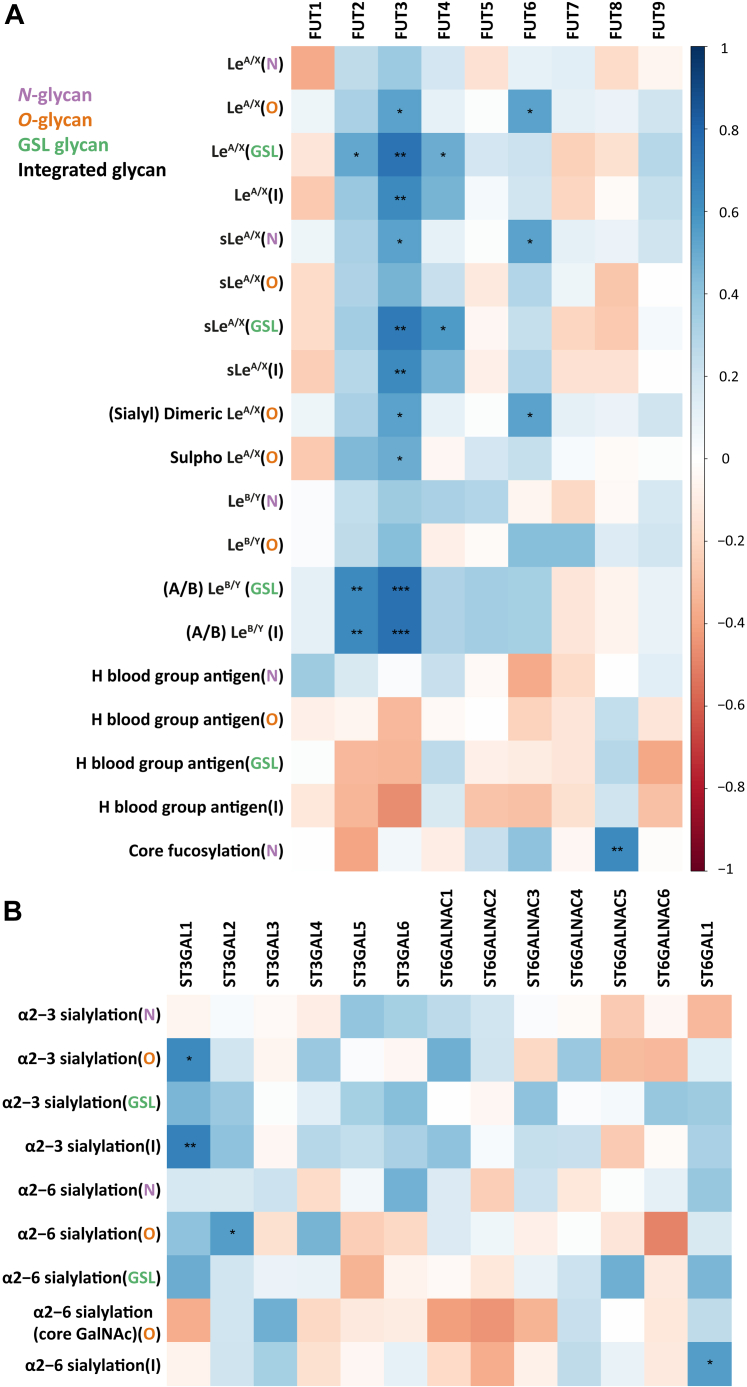
**Correlation of *N*-, *O*-glycans, and GSL glycan features with corresponding GT expression.***A*, correlation of fucosyltransferases with (s)Le and H blood group antigens. *B*, correlation of sialylation with corresponding GTs. The correlation analysis was performed on the basis of the relative quantification of glycosylation features and expression of relevant GTs with the Spearman method. Significant values are marked with ∗ (*p* ≤ 0.05), ∗∗ (*p* ≤ 0.01), and ∗∗∗ (*p* ≤ 0.001). GSL, glycosphingolipid; GT, glycosyltransferase.

To explore the underlying pathways that regulate the glycosylation features of glycans in CRC, correlations between glycosylation features and expression of GTs were explored by Spearman correlation (supplemental Table S8). GT FUT2, encoded by the gene *fucosyltransferase 2* and catalyzing the transfer of L-fucose to the terminal galactose of both *N*- and *O*-glycan and GSL glycans *via* α1-2 linkage (32, 33), significantly correlated with (A/B) Le^B/Y^ on GSL glycans and integrated (A/B) Le^B/Y^ (which was calculated by summing the (A/B) Le^B/Y^ glycosylation traits of *N*-, *O*-glycan, and GSL glycans) (Fig. 3*A*). Surprisingly, positive correlation was also found between FUT2 and Le^A/X^ on GSL glycans which was unexpected as FUT2 does not catalyze the biosynthesis of Le^A/X^ structures. GT FUT3, responsible for catalyzing the transfer of L-fucose to Galβ1-4/3GlcNAc of glycans *via* α1-3/4 linkage to form Le^A/X/B/Y^ antigens and sLe^A^ and disialyl Le^A^ structures (25, 34, 35, 36), positively correlated with Le^A/X^ on *O*-glycan, GSL, and integrated glycans, sLe^A/X^ on *N*-glycans, GSL, and integrated glycans, (s)dimeric Le^A/X^ and sulfo Le^A/X^ on *O*-glycans as well as (A/B) Le^B/Y^ on GSL glycans and integrated glycans (Fig. 3*A*). GT FUT4, involved in the biosynthesis of (s)Le^X^ antigens by transferring L-fucose to GlcNAc of type 2 *N*-acetyllactosamine in α1-3 linkage (37, 38, 39), positively correlated with (s)Le^A/X^ antigen on GSL glycans (Fig. 3*A*). GT FUT5, also reported to be responsible for the synthesis of (s)Le^X^ antigens (40, 41, 42), showed no significant correlation with (s)Le^X^ antigens (Fig. 3*A*). Expression of GT FUT6 participating in the formation of (sialyl) Lewis antigens (43, 44) positively correlated with Le^A/X^ and (sialyl) dimeric Le^A/X^ on *O*-glycans as well as sLe^A/X^ on *N*-glycans. Expectedly, FUT8, which catalyzes the addition of fucose to first GlcNAc of *N*-glycans *via* α1-6 linkage (45), correlated with core fucosylation on *N*-glycans (Fig. 3*A*).

ST3GAL1, participating in the biosynthesis of terminal sialylation of glycoproteins and glycolipids in α2-3 linkage (46), positively correlated with α2-3 sialylation on *O*-glycans and integrated α2-3 sialylation (Fig. 3*B*). In contrast, no positive correlation was found between ST3GAL3/4/6 and α2-3 sialylation in all three glycan classes (Fig. 3*B*), which was somewhat unexpected as these enzymes have been reported to be involved in the synthesis of α2-3 sialylation on glycoprotein and glycolipids (47, 48). ST3GAL2, which has been reported to be primarily involved in the α2-3 sialylation of ganglio and globo series glycolipids (49, 50), showed no significant correlation with α2-3 sialylation of GSL glycans but unexpectedly showed a positive correlation with α2-6 sialylation on *O*-glycans (Fig. 3*B*). Expectedly, ST3GAL5 responsible for the addition of sialic acid to lactosyl ceramide forming gangliosides (51) was found to be positively correlated to α2-3 sialylation on GSL glycans (Fig. 3*B*).

ST6GALNAC1-4 is responsible for adding sialic acid to GalNAc of *O*-glycans (24) yet showed no significant correlation with α2-6 sialylation on *O*-glycans. ST6GALNAC5/6 are known to transfer sialic acid to glycolipids forming α2-6 linkage (25, 26), and also here, no positive correlation was found with α2-6 sialylation on GSL glycans (Fig. 3*B*). ST6GAL1, responsible for transferring a sialic acid to galactose-containing acceptor substrates mainly on *N*-glycans and GSL glycans (52, 53), showed no positive correlation with α2-6-sialylation on *N*-glycans and GSL glycans individually but significantly correlated with integrated α2-6 sialylation (Fig. 3*B*).

### Association of Transcription Factors With GTs and Glycosylation Features in CRC Cell Lines

To obtain insights into the potential regulation of the expression of glycosylation features, the TFs with the highest difference in expression between colon-like and undifferentiated cell lines were probed for association with glycans and GTs (23). Elevated expression of TFs *CDX1, ETS2, HNF1A, HNF4A, MECOM,* and *MYB* has been found for colon-like cell lines whereas the increased expression of TFs *MLLT10*, *MSX1, SIX4, ZNF286A,* and *ZNF286B* have been observed in undifferentiated cell lines (23). Correlations between TFs, GTs, and corresponding glycosylation features were assessed with the Spearman method (supplemental Table S9).

**Fig. 4:**
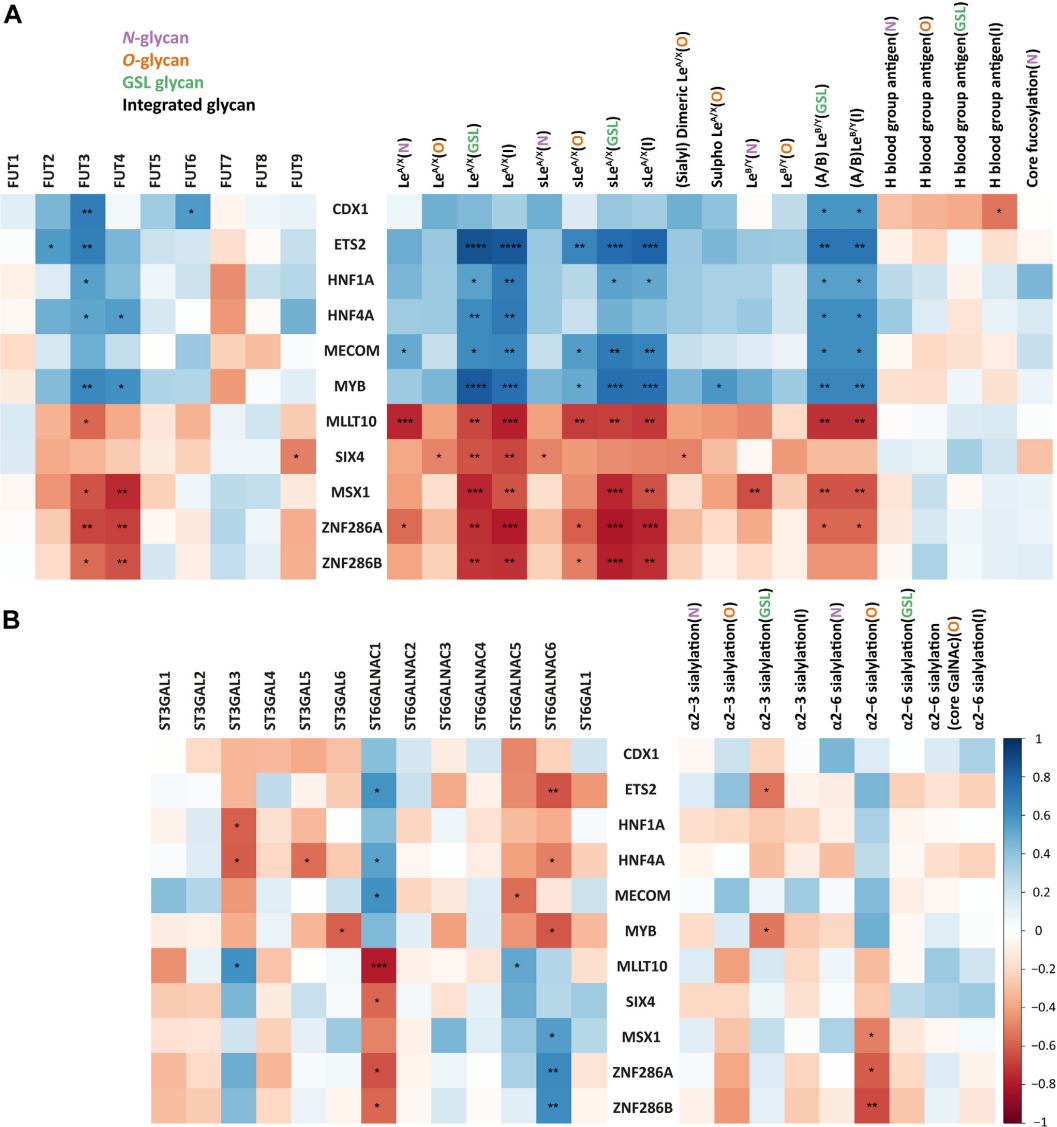
**Correlation of glycosylation features of *N*-, *O*-glycans, and GSL glycans with the transcriptomics of selected TFs in CRC cell lines.***A*, correlation of TFs with fucosyltransferase, (s)Le, and H blood group antigens. *B*, correlation of TFs with sialylation and corresponding GTs. The Spearman method was applied for the correlation analysis between the expression of TFs and glycosylation features. Significant values are marked with ∗ (*p* ≤ 0.05), ∗∗ (*p* ≤ 0.01), ∗∗∗ (*p* ≤ 0.001) and ∗∗∗∗ (*p* ≤ 0.0001). CRC, colorectal cancer; GSL, glycosphingolipid; GT, glycosyltransferase; TF, transcription factor.

The correlation heatmap illustrates that *FUT3* correlates with TFs *CDX1, ETS2, HNF1A, HNF4A,* and *MYB* which in turn correlated with (A/B) Le^B/Y^ (GSL glycans) and integrated (A/B) Le^B/Y^ (Fig. 4*A*). Next to *FUT3*, also *FUT6* positively correlated with TF *CDX1*. *FUT4* exhibited significantly positive correlations with *HNF4A* and *MYB*. Interestingly, Le^A/X^ on GSL glycans and integrated Le^A/X^ showed distinct positive corrections with *ETS2, HNF1A, HNF4A, MECOM,* and *MYB* but negative correlations with *MLLT10, MSX1, SIX4, ZNF286A,* and *ZNF286B* which, as previously mentioned, was highly expressed in undifferentiated cell lines. TF *MECOM* showed positive correlation with Le^A/X^ on *N*-glycans (Fig. 4*A*). Additionally, *ETS2, MECOM,* and *MYB* show significant correlations with sLe^A/X^ (*O*-glycan, GSL, and integrated glycans) which negatively correlated with *MLLT10, ZNF286A,* and *ZNF286B* (Fig. 4*A*). *CDX1* exhibited significant negative correlation with integrated H blood group antigens (Fig. 4*A*).

Regarding the association of sialyltransferases with TFs, *ST6GALNAC1* positively correlated with TF *ETS2, HNF4A,* and *MECOM*, whereas negative correlations were found between *ST6GALNAC6* and *ETS2, HNF4A,* and *MYB* (Fig. 4*B*). TFs *MSX1, ZNF286A,* and *ZNF286B* showed negative correlations with α2-6 sialylation on *O*-glycans and accordingly with *ST6GALNAC1*, while these TFs showed a positive correlation with *ST6GALNAC6* (Fig. 4*B*).

## Discussion

We recently performed an in-depth *N*-, *O-*glycomic, and GSL glycomic analyses of different CRC cell lines revealing commonalities as well as striking diversities of glycosylation (20, 21, 23). To reveal commonalities and discrepancies of expression patterns of glycosylation features shared between the three glycomics layers of CRC cell lines and to explore how these glycosylation features contribute to the molecular differentiation pattern of CRC cell lines, we integrated the available data and assigned them to the different glycosylation features (supplemental Tables S2–S5). A clear separation of CRC classifications was revealed driven by the glycosylation features across the three glycan layers with high abundance of (s)Le^A/X^ antigens in colon-like cell lines (Fig. 1), especially for cell line LS174T and its parent cell line LS180 (54). A previous study suggested that two clonal cell lines, LSB expressing only the truncated CA Tn (GalNAcα-Ser/Thr) and sialyl-Tn on their mucin molecules and LSC with elongated oligosaccharide chains, derived from LS174T cell line (55), which might result from the genetic variation and differences in epigenetic signatures. Due to its natural heterogeneity, cell line LS174T might be considered as representative model of primary CRC tumor.

The sLe^A/X^ antigens in glycoproteins and glycolipids are typical tumor-associated CAs and are involved in tumor progression (4, 5, 56, 57). Interestingly, particularly high expression of (s)Le^A/X^ antigens was found in cell line SW1116 in which sLe^A^, also known as CA19-9, was first discovered with a mouse monoclonal antibody (1116-NS-19-9) (58, 59, 60, 61). Nowadays, CA19-9 is used as serum diagnostic biomarker for CRC and treatment monitoring and associates with poor prognosis (16, 17, 18). Moreover, the expression level of sLe^A^ shows associations with CRC prognosis, specifically the incidence of recurrence and the survival time (62, 63, 64). Elevated levels of sLe^A^ have been reported for *N*-, *O*-glycans, and GSL glycans in CRC (65, 66, 67) which is in line with our results for colon-like cell lines that showed consistent high expression of (s)Le^A/X^ antigen across the three glycan classes (Fig. 2). Taking the previous findings and current results into account, the sLe^A/X^ antigens might be potential targets for treatment of well differentiated CRC. A limitation of the current study is its inability to fully define Le antigens due to the lack of or low abundance of diagnostic ions in tandem MS. Full definition of Le antigens may be achieved by implementing orthogonal approaches such as ion mobility MS. In addition, Tn antigen and other small *O*-glycans were insufficiently covered in our analysis due to the partial loss of small glycans during solid-phase extraction cleanup using PGC self-packed columns. To address this limitation, additional material needed to be investigated for improving solid-phase extraction cleanup.

More than 3 decades ago, the overexpression of Le^Y^ antigen was reported in CRC with detection of monoclonal antibody AH6 and considered to be a diagnostic marker of CRC (68), and the upregulation of Le^B/Y^ antigens has been attributed to poor prognosis of CRC (69). In this study, Le^B/Y^ antigens were detected across the three glycan classes with no significant association between the classes (Fig. 2*A*). Another study demonstrated that transfection of rat CRC cells with cDNA encoding for α1-2 fucosyltransferase promoted the tumorigenicity and enhances cell motility by increased expression of Le^B/Y^ and H blood group antigens (70). Previous research reported that increased expression of FUT4 might be related to upregulation of Le^Y^ in CRC tissues (71); however, according associations were not observed in this study. Interestingly, *FUT2/3* showed a positive correlation with (A/B) Le^B/Y^ antigens on GSL glycans, whereas no correlations were observed between *FUT2/3* and Le^B/Y^ antigens on *N*- and *O*-glycans (Fig. 3*A*), which might suggest GSLs as major substrates of GTs FUT2/3 for biosynthesis of Le^B/Y^ antigens in CRC cell line. FUTs are involved in biosynthesis of Le antigens (25, 34, 35, 39, 72, 73). Regarding FUT3, it not only catalyzes the synthesis of Le^A/X/B/Y^, preferring to type chain 1 over type chain 2 (72), but also participates in the formation of sLe^A^ and disialyl Le^A 25, 34–36^. Significant correlations were discovered between Le^A/X/B/Y^, sLe^A/X^, and (s)dimeric Le^A/X^ with *FUT3* (Fig. 3*A*) which suggests associations with the carcinogenesis of CRC (74). Besides, upregulation of FUT3 is a marker of lower overall survival of breast cancer (75), and knockdown of *FUT3* inhibits the proliferation, migration, tumorigenesis, and TGF-β induced EMT in pancreatic cancer (76). FUT4 contributes to the biosynthesis of (s)Le^X^ (37, 38, 39) which was observed to be positively correlated with (s)Le^A/X^ on GSL glycans but, interestingly, not with *N*- and *O*-glycans (Fig. 3*A*). This is not in line with literature reporting sLe^X^ to be mainly regulated by FUT6 in CRC as well as breast cancer (27, 77). Interestingly, in AML cell lines, we found that (s)Le^A/X^ antigen expression was positively correlated with *FUT7* instead of *FUT3/4* (78). We hypothesize that the biosynthesis of glycans might be regulated by different GTs in a disease- and tissue-specific manner. FUT6 participates in the formation of (s)Le^X^ antigen (43, 44), which positively correlated with Le^A/X^ and (s)dimeric Le^A/X^ on *O*-glycans as well as sLe^A/X^ on *N*-glycans (Fig. 3*A*). Thus, except for the glycosylation features, the corresponding GTs such as FUT3/4/6 might also be promising targets to study the underlying mechanism in the development of CRC.

In addition, the upregulation of H blood group antigens (especially on *O*-glycans and GSL glycans) was found in undifferentiated cell lines (Fig. 2*B*). A previous study demonstrated that H blood group antigens modulate the tumorigenicity of CRC and contributed to the CRC tumor progression (79). Overexpression of H blood group antigen caused by upregulation of α1-2 fucosyltransferase has been shown to associate with poor prognosis in CRC and promote cancer cell mobility (69, 70). Another study indicated α1-2 fucosylation as a predictor of postoperative poor prognosis of CRC (80). Similarly, ABO (H) blood group expression has potential as a prognostic factor for recurrence in ovarian and vulvar cancer (81). In contrast, in bladder tumor, the lack of ABO (H) antigen is a well-documented event and was associated with tumor progression and recurrent disease which are attributed to the loss of relevant GT activities due to downregulation of ABO (H) mRNA transcripts (82, 83, 84). In the present study, although significant correlations were revealed for H blood group antigen between three classes of glycans (*N*-, *O*-glycans, and GSL glycans; Fig. 2*A*), no significant association was observed between H blood group antigen and α1-2 fucosyltransferase FUT1 and FUT2 (Fig. 3*A*). FUT2 rather showed positive correlations with (A/B) Le^B/Y^ on GSL glycans and integrated (A/B) Le^B/Y^ antigens (Fig. 3*A*) which have been reported to be involved in the poor prognosis of CRC (69). In breast cancer, FUT1 and FUT2 have been involved in regulating growth, adhesion, and migration of breast cancer and might serve as a therapeutic target (85). For CRC with undifferentiated stage, the H blood group antigens and relevant GTs may be considered as potential treatment targets.

*CDX1* as a colon-specific TF involved in cell differentiation has been associated with different glycosylation features (86). In our previous study, we discovered that differentiated cell lines expressing *CDX1* featured high multifucosylation and showed a less invasive and less aggressive phenotype (87). The correlation of fucosylation with *CDX1* was revealed in CRC (87, 88), which presents positive correlations with (A/B) Le^B/Y^ on GSL glycans and integrated (A/B)Le^B/Y^ as well as with corresponding GTs FUT3 and FUT6 (Fig. 4*A*). Moreover, we found that TF *ETS2* upregulated in colon-like cell lines significantly correlated with GSL glycans carrying (s)Le^A/X^ and (A/B) Le^B/Y^. *ETS2* has been reported to play critical roles throughout all stages of tumorigenesis and was demonstrated to promote angiogenesis in breast cancer (89, 90). Besides, other TFs like *HNF1A* and *HNF4A* were found to participate in the regulation of antenna fucosylation on *N*-glycans (86) for which no correlation was found in this study. However, positive correlations were found for Le^A/X^ and (A/B) Le^B/Y^ antigens on GSL glycans with *HNF1A* and *HNF4A* as well as with *FUT3*. When exploring the correlation of TF *MYB* with expression of glycosylation features, we found that *MYB* positively associated with (s)Le^A/X^, (A/B) Le^B/Y^ on GSL glycans, sialyl/sulfo Le^A/X^ on *O*-glycans, and their relevant GTs FUT3/4 (Fig. 4*A*). Upregulation of *MYB* has been found to be a predictor for poor prognosis of CRC (91, 92, 93). Taken together, we hypothesize that high expression of TFs (*CDX1, ETS1, HNF1/4A, MECOM,* and *MYB*) might have influence on the overexpression of (s)Le antigens in colon-like cell lines by regulation of their corresponding GT FUT3/4. To prove our hypothesis, more biological experiments are required. In this study, transcriptomic expression of TFs was correlated with MS glycomic data to obtain insights into the potential regulation of glycan expression. The protein expression and activity of these TFs might, however, only poorly correlate with transcript expression. More experiments such as chip-seq data examining the binding of the TFs to the relevant loci need to be conducted in future studies.

In conclusion, the glycosylation patterns across three glycan classes were assessed for CRC cell lines. We revealed overexpression of (s)Le antigens in colon-like cell lines on all three glycan classes and high abundance of H blood group antigens or sialylation in undifferentiated cell lines. The significate correlations observed between (s)Le antigens on three glycan classes with FUT3, partly with FUT4/6, instead of other FUTs indicated that FUT3 may be the main contributor to the biosynthesis of (s)Le antigens in CRC. In addition, FUT3/4 showed stronger correlations with (s)Le antigens on GSL glycans compared to that on *N*- and *O*-glycans, indicating that FUT3/4 might preference for expression of (s)Le antigens on three class glycans. The discovered relationship between upstream TFs with (s)Le antigens and FUT3 indicates that these upstream TFs might contribute to the upregulation of (s)Le antigens *via* regulation of FUT3.

## Data availability

Glycomics data for CRC cell lines were obtained from GlycoPOST: GPST000239 (GSL glycans), GPST000035 (*O*-glycan), and GPST000302 (*N*-glycan). The transcriptomics data of CRC cell lines were retrieved from the Gene Expression Omnibus GSE97023.

## Supplemental Data

This article contains supplemental data (20, 21, 22, 23, 94)

## Conflict of interest

The authors declare no competing interests.
