# Supplementary material for: (Sialyl)Lewis Antigen Expression on Glycosphingolipids, N-, and O-Glycans in Colorectal Cancer Cell Lines is Linked to a Colon-Like Differentiation Program
Source: Mol Cell Proteomics. 2024 Apr 25;23(6):100776. doi: 10.1016/j.mcpro.2024.100776 (PMC11128521; doi:10.1016/j.mcpro.2024.100776)
Supplement: Supplemental Figures S1 and S2 [file mmc2.docx]

**Supporting Information**

(Sialyl)Lewis antigen expression on glycosphingolipids, *N*- and *O*-glycans in colorectal cancer cell lines is linked to a colon-like differentiation program

Di Wang^1^, Katarina Madunić^2^, Oleg A. Mayboroda^1^, Guinevere S.M. Lageveen-Kammeijer^1,3^, and Manfred Wuhrer^1,#^

^1^ Leiden University Medical Center, Center for Proteomics and Metabolomics, Albinusdreef 2, 2333 ZA Leiden, The Netherlands

^2^ Copenhagen Center for Glycomics, Department of Cellular and Molecular Medicine, University of Copenhagen, 2200 Copenhagen, Denmark

^3^ University of Groningen, Groningen Research Institute of Pharmacy, Analytical Biochemistry, 9700 AD Groningen, the Netherlands.

*Corresponding author: [m.wuhrer@lumc.nl](mailto:m.wuhrer@lumc.nl), tel: +31(0)71 526 6989

Contents

[Supplementary Figure 1 2](#_Toc124259578)

[Supplementary Figure 2 3](#_Toc124259579)

# Supplementary Figure 1


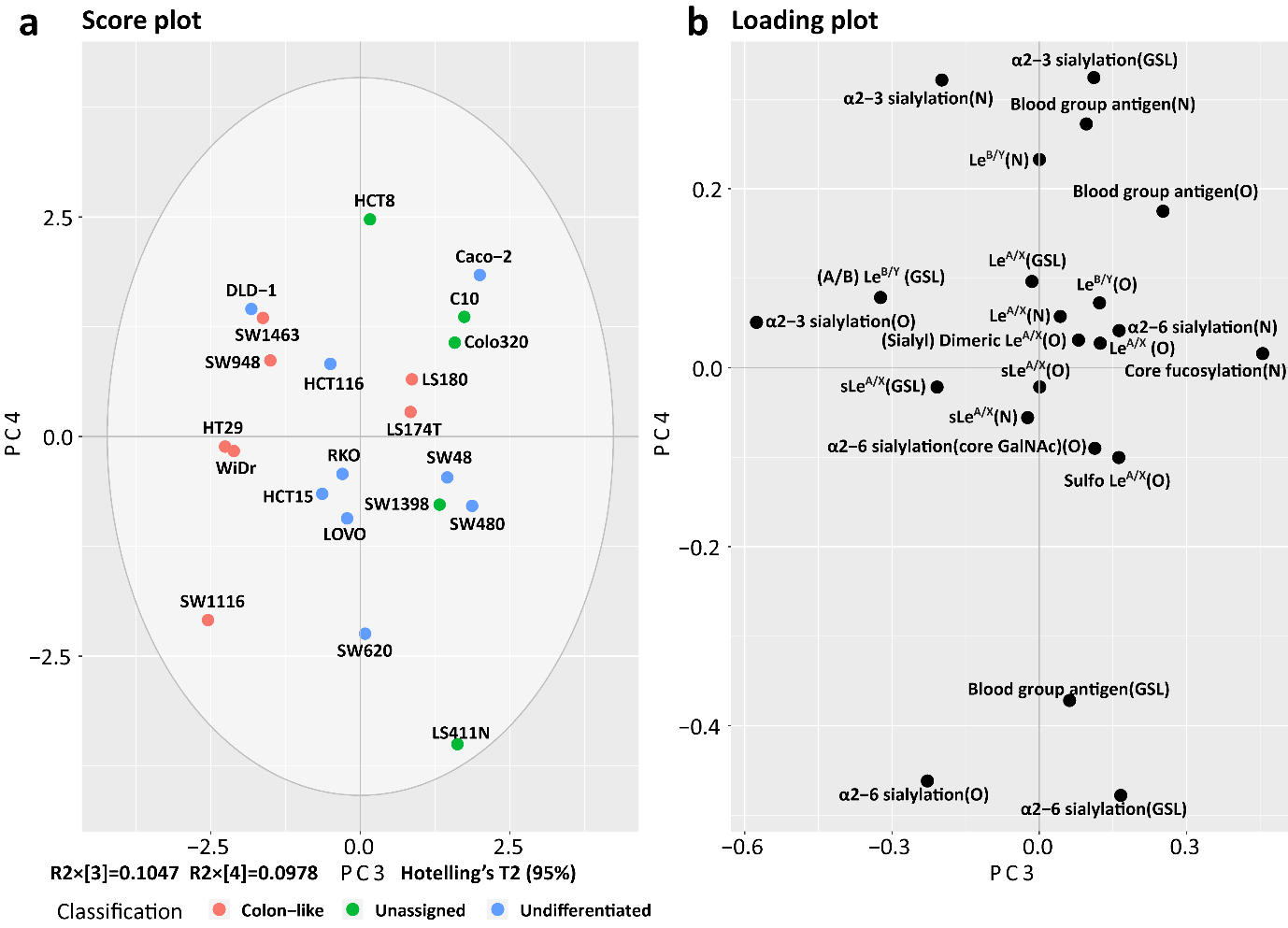


**Supplementary Figure 1**. **Principle component analysis (PCA) of glycosylation features on *N*-, *O*-, and GSL glycans in CRC cell lines.** The third and fourth principal components of the principal component analysis (PCA) model explained 20% of the variance. In total, the first four principle components explain 68.7% of the variance. (**a**) The most prominent separation between different types of CRC cell lines is found between principal components (PC) 3 and 4, driven by the glycosylation features in the (**b**) loading plot.

# Supplementary Figure 2


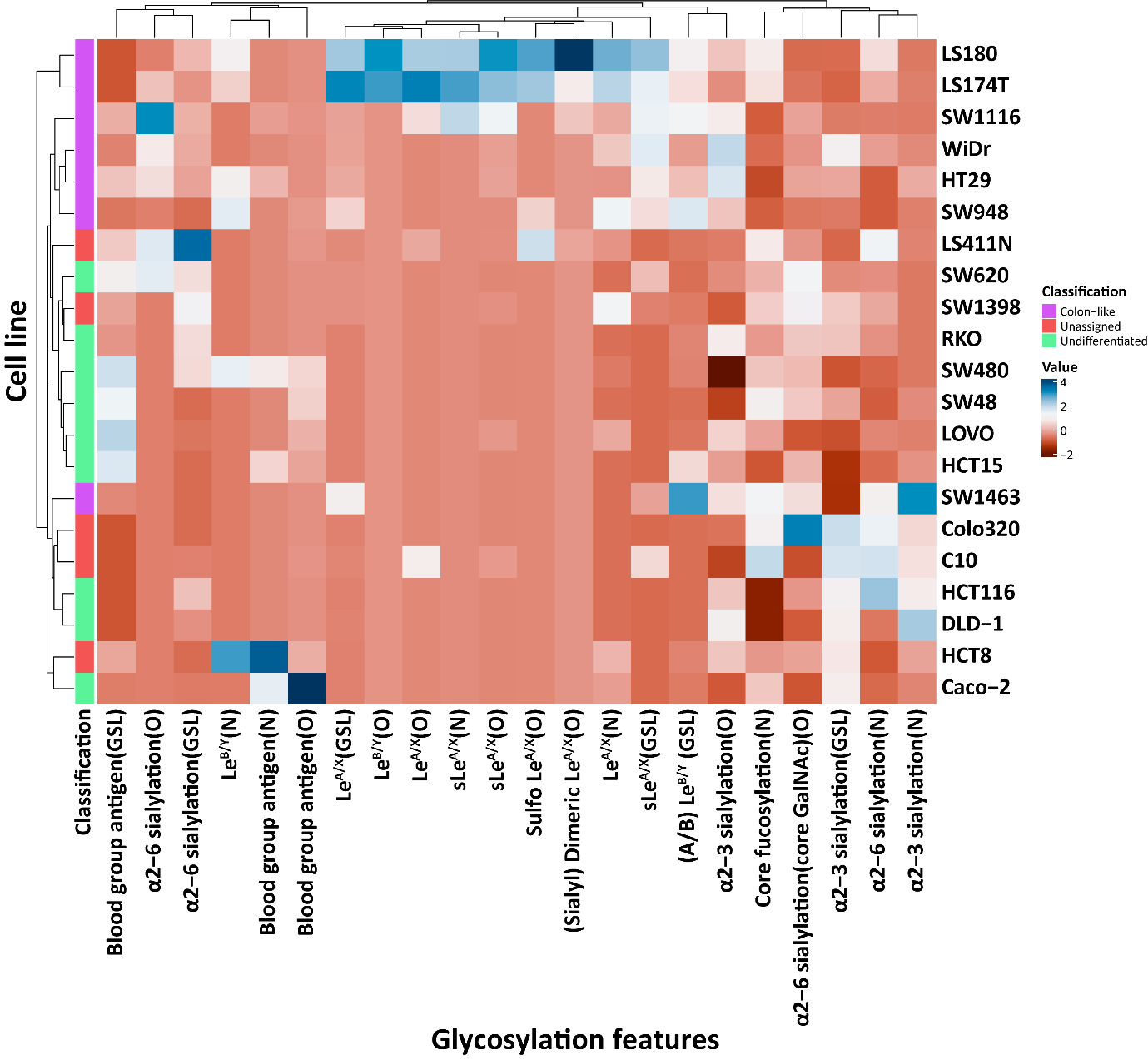


**Supplementary Figure 2**. **The overview of the distribution of glycosylation features on *N*-, *O*-, and GSL glycans (bottom) in CRC cell lines (right).** The relative abundance of each glycosylation feature on *N*-, *O*-, and GSL glycans was calculated. The standardization was performed by taking the mean, equaling it to zero, and the standard deviation was made equal to one. The cell lines were colored on the basis of CRC cell line classification. The composition of Le^B/Y^ A variant is GalNAcα1-3(Fucα1-2)Galβ1-3/4(Fucα1-4/3)GlcNAc-R and for variant B Galα1-3(Fucα1-2)Galβ1-3/4(Fucα1-4/3)GlcNAc-R.
